# Cisplatin-Induced Reproductive Toxicity and Oxidative Stress: Ameliorative Effect of Kinetin

**DOI:** 10.3390/antiox11050863

**Published:** 2022-04-28

**Authors:** Rania Abdel-Latif, Moustafa Fathy, Hend Ali Anwar, Muhammad Naseem, Thomas Dandekar, Eman M. Othman

**Affiliations:** 1 Department of Pharmacology and Toxicology, Faculty of Pharmacy, University of Minia, Minia 61519, Egypt; rania.abdellatief@mu.edu.eg; 2 Department of Biochemistry, Faculty of Pharmacy, University of Minia, Minia 61519, Egypt; mostafa_fathe@mu.edu.eg (M.F.); hendali11996@gmail.com (H.A.A.); 3 Department of Life and Environmental Sciences, College of Natural and Health Sciences, Zayed University, Abu Dhabi 144534, United Arab Emirates; muhammad.naseem@zu.ac.ae; 4 Department of Bioinformatics, Biocenter, Am Hubland University of Wuerzburg, 97074 Wuerzburg, Germany

**Keywords:** cytokinins, kinetin, cisplatin, reproductive toxicity

## Abstract

Cisplatin is a commonly used chemotherapeutic agent; however, its potential side effects, including gonadotoxicity and infertility, are a critical problem. Oxidative stress has been implicated in the pathogenesis of cisplatin-induced testicular dysfunction. We investigated whether kinetin use at different concentrations could alleviate gonadal injury associated with cisplatin treatment, with an exploration of the involvement of its antioxidant capacity. Kinetin was administered in different doses of 0.25, 0.5, and 1 mg/kg, alone or along with cisplatin for 10 days. Cisplatin toxicity was induced via a single IP dose of 7 mg/kg on day four. In a dose-dependent manner, concomitant administration of kinetin with cisplatin significantly restored testicular oxidative stress parameters, corrected the distorted sperm quality parameters and histopathological changes, enhanced levels of serum testosterone and testicular StAR protein expression, as well as reduced the up-regulation of testicular TNF-α, IL-1β, Il-6, and caspase-3, caused by cisplatin. It is worth noting that the testicular protective effect of the highest kinetin dose was comparable/more potent and significantly higher than the effects of vitamin C and the lowest kinetin dose, respectively. Overall, these data indicate that kinetin may offer a promising approach for alleviating cisplatin-induced reproductive toxicity and organ damage, via ameliorating oxidative stress and reducing inflammation and apoptosis.

## 1. Introduction

Cancer is one of the most lethal diseases characterized by uncontrolled cell proliferation and aberrant cell cycle activity driven by genetic and environmental factors [1]. Chemotherapy is the most common and powerful drug treatment, which is used to eliminate fast-growing cells even at distant sites from the origin of the primary tumor through a local or systemic effect [2]. Although the use of chemotherapy significantly increases the cure rate of many malignancies and improves patients’ survival rates, the classical chemotherapeutic drugs are cytotoxic and do not distinguish between cancer and normal cells [3], and as a consequence, the patients develop serious side effects that are considered during the treatment and limit the clinical use [4,5]. Cisplatin (CIS) is a standard and commonly used therapeutic agent in the treatment of various malignant tumors including lung, breast, ovarian, bladder, and testicular cancers [6]. The use of CIS-based chemotherapy is associated with cure rates of up to 90% for the most frequent malignant cancers [7]. For young men of reproductive age, testicular dysfunction and infertility associated with CIS therapy are a major concern [4]. It has been suggested that more than 50% of the patients treated with CIS showed long-term infertility or permanent azoospermia [8]. Besides steroidogenesis suppression and spermatogenesis disruption, CIS-treated patients can also elicit abnormal spermatozoa morphology [9,10]. Such damage to germ cells could predispose for genetic abnormalities, resulting in a higher risk of birth defects [11]. Accordingly, different organizations such as the American Society for Reproductive Medicine (ASRM) and the American Society of Clinical Oncology (ASCO) have released formal recommendations showing the necessity of searching for solutions for fertility preservation, including sperm cryopreservation [12] and, as another approach, the administration of a gonadotrophin-releasing hormone (GnRH) agonist was reported to restore the ability of spermatogonia [13,14].

Various reports showed that spermatogonial stem cell death following CIS exposure is attributed to oxidative stress, which could alter sperm chromatin integrity and DNA methylation [15,16]. Indeed, accumulating data points to increased oxidative stress in CIS-treated animals along with the excessive release of reactive oxygen species (ROS), lipid peroxidation, which ultimately promotes inflammation, and apoptosis in testicular tissue [10,17]. Numerous approaches have been undertaken to reduce or prevent CIS-related testicular dysfunction, including the use of different antioxidants and cytoprotective agents [18]. However, no conclusively protective pharmacological intervention have shown a significant benefit.

Kinetin is an N6-substituted adenine derivative that was initially detected as a secondary product of DNA damage and plant cell extracts [19]. Kinetin is shown to be implicated in various biological functions in both plants and human cells [20]. It has been suggested that kinetin could promote cell division via abbreviating the cell cycle [21,22]. In addition, kinetin stimulates repair enzyme expression and ribosomal RNA transcription [23]. Besides its role in cell growth and division, kinetin has been shown to possess antioxidant and radical-scavenging activity [24,25]. Importantly, kinetin was reported to regulate antioxidant activities, in particular superoxide dismutase (SOD) and catalase enzymes [26,27]. Based on its antioxidant capacity, kinetin has previously been shown to improve the motility, viability, and structural integrity of animal sperm during cryopreservation [24,28]. The reported beneficial effect of kinetin in protecting canine sperm against oxidative damage could suggest it as a possible adjuvant for the prevention and treatment of various spermiotoxicity disorders. However, no previous studies have evaluated the protective effect of kinetin against CIS-induced testicular injury to date. The aim of this study, therefore, was to delineate the possible protective effects of kinetin in CIS-induced testicular toxicity that might be exhibited in different doses and explore the mechanisms that underlie these possible effects, including its potential effect on oxidative stress as a plausible mechanism.

## 2. Materials and Methods

### 2.1. Chemicals

Kinetin was obtained from Sigma-Aldrich (Dorset, Germany). CIS was purchased from the Mylan SAS pharmaceutical company (Saint-Priest, France). Kits for reduced glutathione (GSH), superoxide dismutase (SOD), and catalase were purchased from Biodiagnostic (Cairo, Egypt). Caspase-3 and steroidogenic acute regulatory protein (StAR) polyclonal antibodies were purchased from Santa Cruz Biotechnology (Dallas, TX, USA). Rat ELISA kits of tumor necrosis factor alpha (TNF- α) and interleukin-6 (IL-6) were obtained from Elabscience biotechnology (Houston, TX, USA), while rat testosterone and interleukin-1β(IL-1β) ELISA kits were purchased from Cusabio Technology LLC (Houston, TX, USA) and Wuhan Fine Biotech Co., Ltd. (Wuhan, China), respectively.

### 2.2. Experimental Animals

**Figure 1:**
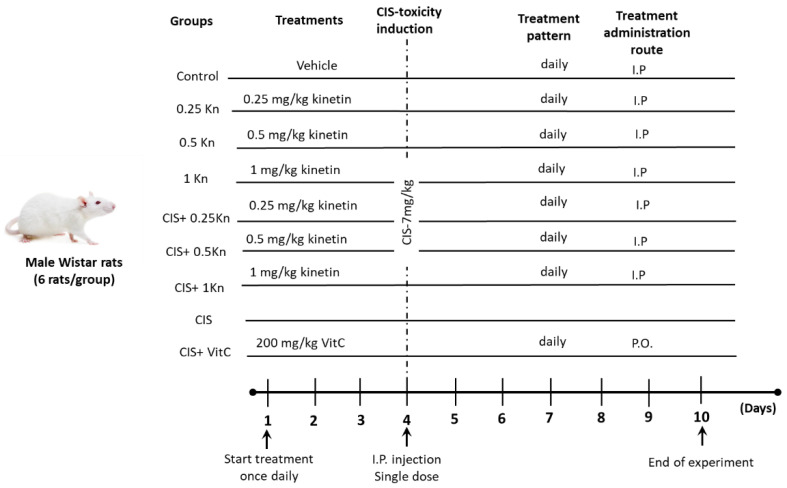
Schematic representation of the experimental design, showing the study groups and the timeline. I.P., intraperitoneal injection; P.O., oral administration; Kn, kinetin; CIS, cisplatin; VitC, vitamin C.

Figure 1 shows the protocol design and pharmacological treatments. Adult male Wistar rats weighing 140–160 g were obtained from the Animal Care Unit, Faculty of Agriculture, Minia University. Animals were housed in the standard animal facility (12 h lighting cycle and 24 ± 2 °C temperature), and were supplied with tap water and standard food. All animals’ procedures were performed in accordance with the Animal Care Community, Minia University, Egypt (Permit Number: 54/2019).

After an acclimatization period of 2 weeks, rats were randomly divided into nine groups (six animals in each): a control group, receiving only the saline vehicle for 10 days; a 0.25 Kn group, receiving a single daily IP dose of 0.25 mg/kg kinetin for 10 days; a 0.5 Kn group, receiving a single daily IP dose of 0.5 mg/kg kinetin for 10 days; a 1 Kn group, receiving a single daily IP dose of 1 mg/kg kinetin for 10 days; a CIS group, receiving a single IP dose of 7 mg/kg cisplatin on the 4th day of the experiment; a CIS+ 0.25 Kn group, receiving a single IP dose of 0.25 mg/kg kinetin daily for 10 days concomitantly with a single IP dose of 7 mg/kg cisplatin on the 4th day of the experiment; a CIS + 0.5 Kn group, receiving a single IP dose of 0.5 mg/kg kinetin daily for 10 days concomitantly with a single IP dose of 7 mg/kg cisplatin on the 4th day of the experiment; a CIS+ 1 Kn group, receiving a single IP dose of 1 mg/kg kinetin daily for 10 days concomitantly with a single IP dose of 7 mg/kg cisplatin on the 4th day of the experiment; and a CIS+ VitC group, receiving a single oral dose of 200 mg/kg vitamin C daily for 10 days concomitantly with a single IP dose of 7 mg/kg cisplatin on the 4th day of the experiment. Kinetin was freshly prepared in saline (0.9%M/V sodium chloride) which was used as a vehicle.

### 2.3. Sample Preparation

At the end of the experimental period of 10 days, the rats were sacrificed, both testes were rapidly excised and blood was withdrawn via the retro-orbital sinus of each rat. For preparation of the serum, blood was allowed to clot by leaving it undisturbed at room temperature for 15–30 min and removal of the clot was carried out by centrifuging at 4500 rpm for 10 min in a refrigerated centrifuge. The resulting supernatant was collected and the serum samples were stored at −80 °C for testosterone analysis.

Rat testes were divided sagittally into two halves; one half was fixed in 10% formalin for histopathological analysis. The other half was snap-frozen in liquid nitrogen and kept at −80 °C for biochemical and Western blot analyses.

For biochemical measurements, testicular tissues were homogenized into 0.05 M phosphate buffer saline (PBS, pH 7.4) then centrifuged at 3000 rpm for 15 min. The supernatant was separated and used for further analysis

### 2.4. Assessment of Sperm Quality

#### 2.4.1. Assessment of Sperm Count

To count epididymal spermatozoa, the epididymis was minced in normal saline, placed in a rocker for 10 min, and incubated at room temperature for 2 min. Then, 2–3 drops of sperm supernatant suspension were transferred to a counting chamber. Then, the number of sperm heads were counted in ten squares under a light microscope at ×200 magnification. The sperm count was recorded as the concentration of spermatozoa (millions of spermatozoa per mL) [29].

#### 2.4.2. Assessment of Sperm Motility

Sperm motility was evaluated by observing a sperm suspension for 3–5 min. The percentage of motile spermatozoa was determined by using the following formula: (mean number of motile spermatozoa/total number of spermatozoa) × 100% [30].

#### 2.4.3. Assessment of Sperm Abnormality

To determine the percentage of morphologically abnormal spermatozoa, the slides stained with eosin–nigrosine were prepared. The slides were then viewed under a light microscope at 200× magnification. A total of 300 sperm cells were examined on each slide (2100 cells in each group) and the head, tail, and total abnormality rates of spermatozoa were expressed as a percentage [29,30].

### 2.5. Histopathological Examination

Testes tissues were fixed in 10% buffered formalin and then embedded in paraffin blocks. Using microtome, paraffin blocks were sectioned at 4 μm thickness, deparaffinized, and stained with hematoxylin–eosin (H&E) for histological examination by light microscope at a magnification of ×200.

### 2.6. Biochemical Analysis

#### 2.6.1. Determination of Serum Testosterone Level

Serum testosterone level was measured in serum using the rat testosterone ELISA kit (Cusabio Technology, Houston, TX, USA) (CAT No: E-EL-R0019), according to the manufacturer’s instructions.

#### 2.6.2. Determination of Testicular Oxidative Stress Biomarkers

Assessment of testicular antioxidant status was carried out in testicular homogenate by evaluating GSH concentration, lipid peroxide content, and levels of catalase and SOD activities. Testicular lipid peroxidation was determined as a thiobarbituric-acid-reacting substance and expressed as equivalents of MDA in nmol/g tissue [31].

GSH level was chemically assessed using a method described previously [32]. The method is based on the sulfhydryl component of GSH reacting with 5,5-dithio-bis-2-nitrobenzoic acid producing a yellow-colored 5-thio-2-nitrobenzoic acid, which was measured colorimetrically at 412 nm, and the results were expressed as nmol/g tissue. SOD enzyme activity was detected colorimetrically at 560 nm and the results were expressed as U/g tissue. The assessment relies on the ability of the SOD enzyme to inhibit the phenazine-methosulphate-mediated reduction of nitroblue tetrazolium dye [33]. Assessment of catalase antioxidant enzymatic activity was determined in testicular homogenates by a colorimetric kit according to the manufacturer’s instructions. The results were expressed as unit/g tissue protein.

#### 2.6.3. Determination of Testicular Inflammation

TNF-α, LI-1β, and IL-6 levels were assessed in the testicular tissue according to the manufacturer’s instructions.

### 2.7. Western Blot Analysis

Western blot analysis was used to detect the protein expression of the steroidogenic acute regulatory protein (StAR) and caspase-3 in testicular tissues. For caspase-3 detection, the testicular tissues were solubilized in RIBA lysis buffer (Bio BASIC INC, Markham Canada) for protein extraction. For StAR protein analysis, a crude mitochondrial preparation was acquired, as described in a previous study [34]. Testis tissue was thoroughly washed in 2 mL of sucrose buffer (0.25 M sucrose, 10 mM tris, 0.1 mM ethylenediaminetetraacetic acid (EDTA), pH 7.4), and the homogenate was centrifuged at 600 rpm for 15 min. The cloudy supernatant, containing the mitochondria, was removed and transferred to another tube, which was then centrifuged at 12,000 rpm for 15 min. The resulting mitochondrial pellet was washed in a sucrose buffer and recentrifuged at 12,000 rpm for 15 min. Then, the pellet was resuspended in a sucrose buffer, and the protein content was determined by using the Bradford assay for both lysates.

The samples (to detect either caspase-3 or StAR protein expressions) were electrophoresed on a 10% sodium dodecyl sulfate gel, then transferred on to a polyvinylidene fluoride (PVDF) membrane by using the Trans-Blot^®^ SD semi-dry electrophoretic transfer cell (Bio-Rad, Hercules, CA, USA). Blocking of the membrane was performed to prevent non-specific background binding with 10% skim dry milk in PBS in Tris-buffered saline at room temperature for 1 h. Membranes were incubated at 4 °C overnight with primary antibodies StAR (1:000) or caspase-3 (1:500). The membrane was washed then incubated with horseradish peroxidase-conjugated secondary antibody for 1 h. The blots were developed using the chemiluminescent method and signals were captured. The bands’ densities were normalized to the corresponding density of the β-actin band and presented as a ratio of the relative optical density.

### 2.8. Statistical Analysis

Data are represented as the mean ± SEM. Differences in statistical significance were evaluated using a one-way analysis of variance (one-way ANOVA test) followed by a Tukey-Kramer post-analysis test for comparing groups. Statistical significance was presented at *p* < 0.05. Analysis was performed using GraphPad Prism^®^ software Inc (Version 8.1.), San Diego, CA, USA.

## 3. Results

### 3.1. Effect on Sperm Quality Assessment

**Figure 2:**
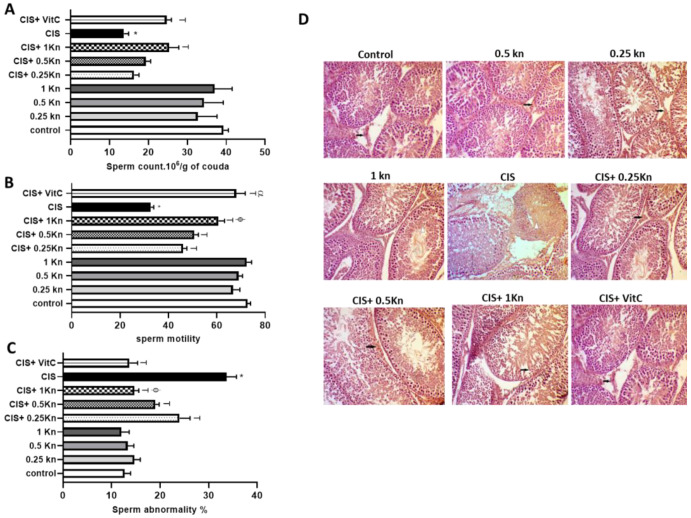
Effect of different doses of kinetin and vitamin C on (**A**) sperm count, (**B**) sperm motility, (**C**) sperm abnormality, and (**D**) histopathological changes in cisplatin treated rats (×200). Data are represented as mean ± SEM, *,T, ф and α are significant differences from control, 0.25 Kn, CIS, CIS+ 0.25 Kn and CIS+ 1 Kn groups, respectively, at *p* < 0.05 where *n* = 5–6. Kn, kinetin; CIS, cisplatin; VitC, vitamin C.

The sperm count analysis (Figure 2A), motility analysis (Figure 2B), and sperm morphology evaluation (Figure 2C) showed that CIS treatment is associated with a significant decrease in sperm count and motion, along with a significant increase in the percentage of abnormal spermatozoa compared with the control group. Compared to CIS-treated rats, an increase in both sperm motility, along with a decrease in the frequency of sperm abnormality was observed in a dose-dependent manner with kinetin concomitant treatment in rats challenged with CIS.

It was observed that only the highest dose of kinetin (1 mg/kg) could significantly protect against the decrease in sperm count in CIS-treated rats, while concomitant treatment of CIS with either 0.25 or 0.5 mg kinetin showed no significant effect on sperm count compared to rats challenged with CIS alone. Concomitant treatment with vitamin C with CIS showed a significant increase in sperm count and motility and a decrease in the percentage of sperm abnormality compared with rats treated with CIS alone. The effect of vitamin C was comparable to the effect of kinetin 1 mg/kg in CIS-treated rats in terms of increasing sperm count and decreasing the frequency of sperm abnormality. However, the CIS+ VitC group showed significantly higher sperm motility than that was observed in the CIS+ 1 Kn group.

Histological examination of the testes is depicted in Figure 2D, showing a normal arrangement of the germinal cells, Sertoli cells, and Leydig cells without histopathological lesions in the control, 0.25 Kn, 0.5 Kn and 1 Kn groups. In contrast, the CIS-treated group showed a distortion of normal testicular architecture as evidenced by spermatogenic cells degeneration, seminiferous tubules atrophy, and tubular hemorrhage. Administration of either vitamin C or kinetin in different concentrations of 0.25, 0.5, and 1 mg/kg along with CIS caused an improvement in the morphology of the seminiferous tubules and germ cells.

### 3.2. Effect on Serum Testosterone and StAR Protein Level

**Figure 3:**
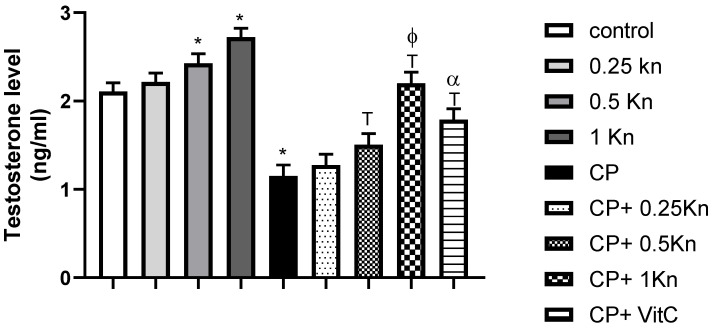
Effect of different doses of kinetin and vitamin C on serum testosterone level in cisplatin-treated rats. Data are represented as mean ± SEM, *, T, ф and α are significant differences from control, 0.25 Kn, CIS, CIS+ 0.25 Kn and CIS+ 1 Kn groups, respectively, at *p* < 0.05 where *n* = 5–6. Kn, kinetin; CIS, cisplatin; VitC, vitamin C.

**Figure 4:**
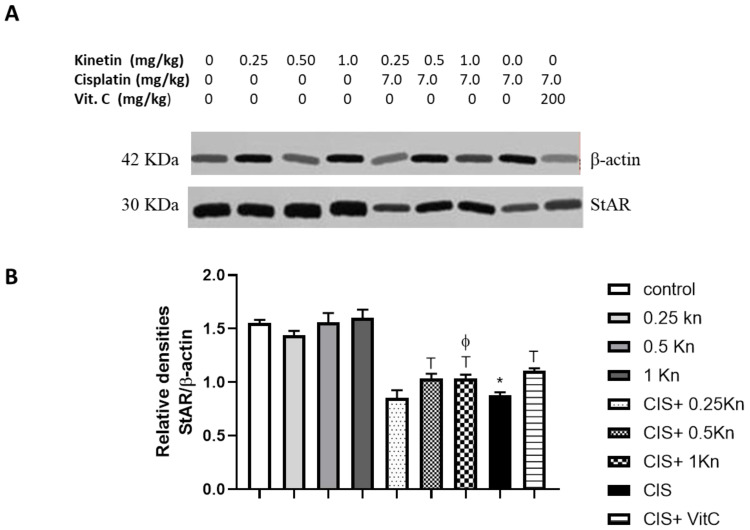
Representative Western blot analysis of StAR protein expression of testicular tissues, showing protein bands of each group (**A**) and graphs presenting their densitometric analysis (**B**). Data are represented as mean ± SEM, *, T, ф and α are significant differences from control, 0.25 Kn, CIS, CIS+ 0.25 Kn and CIS+ 1 Kn groups, respectively, at *p* < 0.05 where *n* = 5–6. Kn, kinetin; CIS, cisplatin; VitC, vitamin C.

The capacity of testosterone synthesis is an important factor that can affect serum testosterone levels [35]. In this regard, we detected serum testosterone and the testicular StAR protein level, which is a rate-limiting step in steroid synthesis. A significant reduction in the serum testosterone concentration and StAR protein level was observed in the CIS-treated group compared to the control group (1.15 ± 0.13 vs. 2.11 ± 0.05 ng/mL, and 0.8793 ± 0.02 vs. 1.55 ± 0.02, respectively) (Figure 3 and Figure 4). However, kinetin administration at doses of 0.5 and 1 mg/kg caused a significant increase in both the serum testosterone level and StAR protein level compared to the CIS-treated group. Notably, administration of the highest kinetin dose (1 mg/kg) showed a significant increase in the testosterone level in CIS-treated rats compared to the effect of a kinetin dose of 0.25 mg/kg.

Although Vitamin C treatment significantly prevented the drop in serum testosterone level in CIS-treated rats, its preventive effect was significantly lower than the effect of the kinetin high dose (1 mg/kg) on testosterone levels in CIS-treated rats. Similarly, Vitamin C showed a significant increase in StAR proteins in CIS-treated rats. However, this effect was comparable to the effect of 1 mg/kg kinetin in CIS-treated rats. Interestingly, rats treated with a dose of kinetin 1 mg/kg alone showed a significant elevation of testosterone levels compared to the control group.

### 3.3. Effect on Testicular Oxidative Stress Parameters

For assessment of testicular oxidative stress status, GSH concentration, catalase and SOD activities, and lipid peroxide content were evaluated in the testicular tissue homogenate. Treatment with CIS caused a significant decrease in testicular GSH concentration, as well as catalase and SOD activities, compared with the control (Table 1). Concomitant treatment of CIS with different concentrations of kinetin (0.25, 0.5, and 1 mg/kg) significantly restored testicular GSH concentration and activities of catalase and SOD, compared to the CIS-treated group. Notably, the up-regulation of antioxidant enzymes by 1 mg of kinetin in the CIS-treated group was significantly higher than that produced by the lowest dose of kinetin in CIS-treated rats. Although vitamin C treatment in CIS-treated rats showed a significant increase in GSH concentrations and activities of SOD and catalase, this effect was significantly lower than that produced by 1 mg/kg kinetin in CIS-treated rats.

CIS significantly increased MDA as an indicator for testicular lipid peroxidation control (Table 1). Concomitant administration with either vitamin C or different kinetin concentrations (0.25, 0.5, and 1 mg/kg) restored MDA levels in a statistically significant manner compared to CIS treatment alone. Although the protective effect of a higher dose (1 mg/kg) of kinetin on increased MDA in CIS-treated rats was significantly higher than that produced by 0.25 mg kinetin, concomitant use of vitamin C produced a significant reduction in MDA levels in CIS-challenged rats compared to the CIS+ l Kn group. Interestingly, administration of kinetin alone in different concentrations of 0.25, 0.5, and 1 mg/kg showed a significant antioxidant effect, in terms of increased antioxidant enzymes and decreased MDA levels, compared to the control group. The observed antioxidant effect of kinetin was dose-dependent. Rats treated with a high kinetin concentration of 1 mg/kg showed a significant increase in antioxidant enzymes and a decreased MDA level compared to 0.25 mg/kg kinetin-treated rats.

### 3.4. Effect on Testicular Inflammation

**Figure 5:**
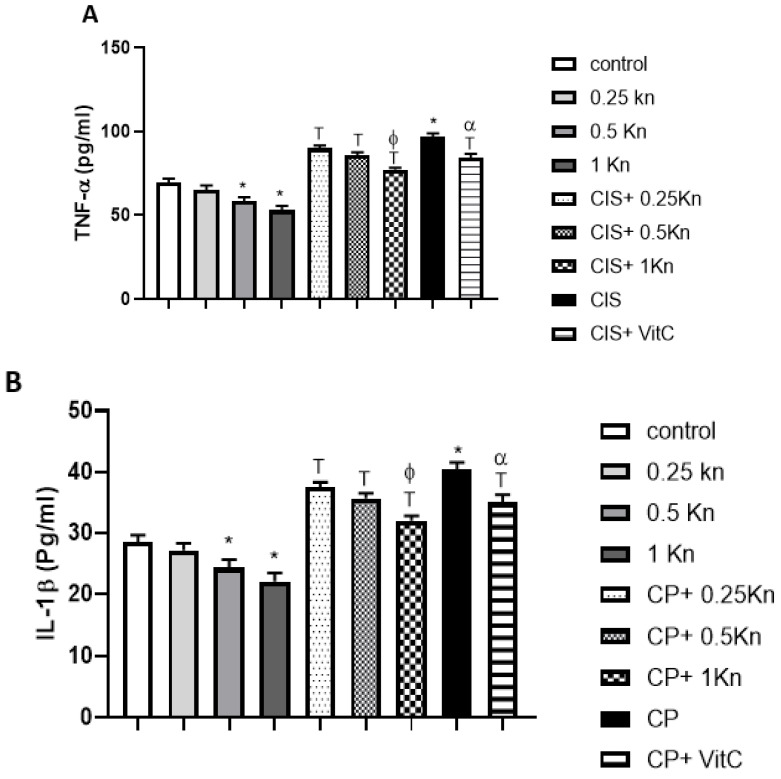

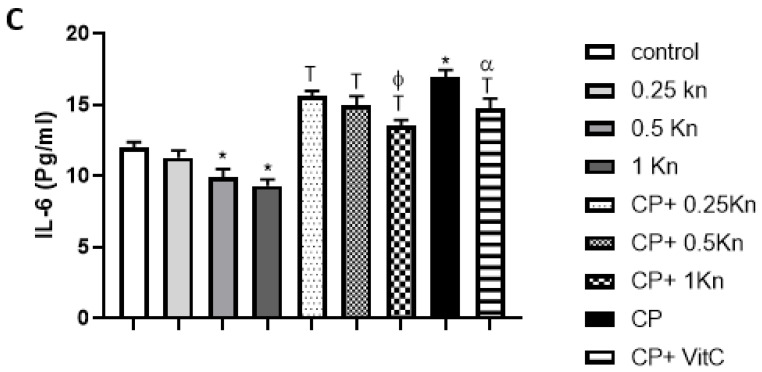
Effect of different doses of kinetin and vitamin C on testicular TNF- α (**A**), IL-1β (**B**) and LI-6 (**C**) in cisplatin-treated rats. Data are represented as mean ± SEM, *,T, ф and α are significant differences from control, 0.25 Kn, CIS, CIS+ 0.25 Kn and CIS+ 1 Kn groups, respectively, at *p* < 0.05 where *n* = 5–6. Kn, kinetin;, CIS, cisplatin; VitC, vitamin C.

Figure 5 depicts a significant increase in testicular TNF-α level as a marker of inflammation in the CIS group compared to the control group. Similarly, levels of IL-1β and IL-6 were significantly higher in the CIS group compared to the control group. Administration of either kinetin in different concentrations of 0.25, 0.5 or 1 mg/kg, or vitamin C, caused significant protection against the up-regulation in testicular TNF-α, IL-1β and IL-6 levels of CIS-treated rats. The anti-inflammatory effect of kinetin at a dose of 1 mg/kg was significantly more potent than the effects of both 0.25 mg/kg kinetin and vitamin C treatment administered in rats challenged with CIS. Interestingly, administration of kinetin alone at doses 0.5 and 1 mg/kg showed a significant decrease in the inflammatory biomarker levels of TNF- α, IL-1β and IL-6 in testicular tissue compared to the control group.

### 3.5. Effect on Testicular Caspase-3 Expression

**Figure 6:**
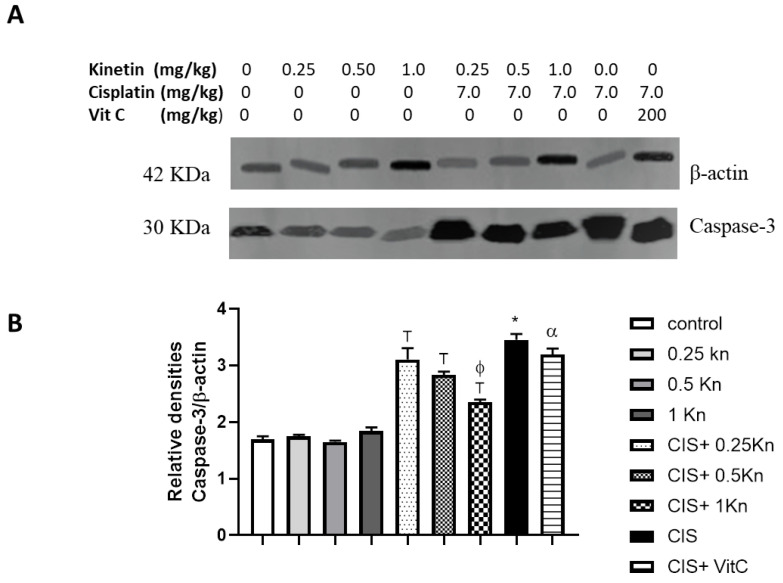
Representative Western blot analysis of caspase-3 protein expression of testicular tissues, showing protein bands of each group (**A**) and graphs presenting their densitometric analysis (**B**). Data are represented as mean ± SEM, *, T, ф and α are significant differences from control, 0.25 Kn, CIS, CIS+ 0.25 Kn and CIS+ 1 Kn groups, respectively, at *p* < 0.05 where *n* = 5–6. Kn, kinetin; CIS, cisplatin; VitC, vitamin C.

Western blot analysis of caspase-3 antibodies as an apoptotic marker and a semi-quantitative analysis were performed in the testicular homogenate of the study groups (Figure 6). While control and kinetin groups (0.25 Kn, 0.5 Kn, and 1 Kn) showed nearly comparable caspase-3 expressions, CIS treatment caused a significant increase in the expression of caspase-3 compared to the control group. Concomitant administration of kinetin in different concentrations along with CIS, significantly protects against increased caspase-3 expression in rats’ testes and the observed protection was dose-dependent. Administration of kinetin 1 mg/kg in CIS-treated rats showed a potent antiapoptotic effect evident by a significant decrease in caspase-3 expression compared to CIS+ 0.25 Kn and CIS+ Vit C.

## 4. Discussion

Testicular injury is a serious adverse effect of CIS therapy, that may result in temporary or permanent gonadal damage in men [36]. Gonadotoxicity following CIS treatment has a significant impact on a patient’s fertility which is of particular concern to cancer patients of a reproductive age [37]. In order to enhance the quality of life of cancer survivors, the preservation of germinal cells is an essential target during CIS chemotherapy [38]. In line with previous reports [39,40], CIS treatment in the current study was associated with a significant decrease in sperm count, sperm motility, and an increase in sperm abnormality rates in rat testes.

Germinal epithelial atrophy leading to azo- or oligospermia has been a recognized consequence of CIS treatment, and it is also associated with abnormalities in the Leydig cells [36], and suppression of testosterone production [40,41]. In the current study, damage in the testicular structure was observed in rats after the administration of CIS at a dose of 7 mg/kg, compared with the control group. CIS-induced testicular damage was characterized by inhibition of testosterone production and StAR protein expression, which is a rate-limiting step in steroid biosynthesis. Although the CIS-related side effect of gonadal toxicity is multifactorial, oxidative stress was reported as one of its major contributors. CIS-induced oxidative stress contributes to male infertility by reducing sperm function [39]. CIS treatment was reported to generate an excessive amount of ROS, which depleted antioxidant activities and increased lipid peroxidation in rat testes [42,43,44]. In agreement with the previous studies, we have reported an impairment in testicular oxidative status of the rat group treated with CIS, which is associated with decreased sperm quality in this group. Indeed, the present investigation showed a significant increase in MDA concentrations in rat testes, along with decreased GSH levels and activities of SOD and catalase. Concomitant treatment with kinetin, however, prevented the CIS-induced testicular atrophy, as shown by counteracting oxidative stress and preserving sperm quality, testosterone level, and StAR protein expression.

A study by Hashem et al. (2018) showed that kinetin has a significant protective effect on improving sperm motility and viability in ram semen, as well as improving antioxidant parameters [24]. However, the mechanism of kinetin’s effect is still unclear. This may be due, in part, to the antioxidant effect of kinetin [25]. Due to the presence of kinetin bases in DNA, it has been reported that kinetin might mediate its antioxidant effect via stimulation of repair enzymes which ultimately prevent oxidative-stress-related DNA damage in spermatozoa [45]. Olsen et al. (1999) explained the DNA protective effects of kinetin against oxidative damage by its ability to prevent ROS formation and scavenge them before they react with the DNA [23]. In the current study, administration of kinetin along with CIS showed protection against oxidative stress compared to the CIS group. The protection against lipid peroxidation and depletion of antioxidant enzymes by kinetin may indicate a possible mechanism for the antioxidative activity of kinetin. Our observations are in line with previous findings that showed the effect of kinetin in reducing the amount of MDA and up-regulating antioxidant enzymes in spermatozoa and different mammalian cells [24,25]. Interestingly, we noticed that kinetin exhibited an antioxidant effect when administered alone or in combination with CIS and this effect was dose-dependent. In this regard, the highest concentration of kinetin (1 mg/kg) showed a greater antioxidant potential than the lowest kinetin concentration of 0.25 mg/kg. Consequently, CIS-treated rats administered the dose of 1 mg/kg kinetin showed a significant increase in sperm quality and testosterone levels, compared to those who received 0.25 mg/kg kinetin along with CIS, and this effect is comparable to or even more significant than the effect of the potent antioxidant vitamin C. The same pattern of results were observed in the context of the StAR protein, the protein that is important for transferring cholesterol [46,47] from the outer to the inner mitochondrial membrane to synthesize testosterone [48], where treatment with kinetin attenuated the reduction in the StAR level upon treatment with cisplatin. The same effect for the cisplatin was reported by Soni et.al (2016), who showed that the StAR level reduced in Sprague Dawley rats if they were treated with cisplatin in a dose-dependent manner [48]. Reduction in the StAR level may explain the observed change in the testosterone level, in addition to testicular injury. In agreement with the current study, Naseem et al. (2020) showed that kinetin protects against oxidative stress in mammalian cellular systems in a dose-dependent manner [49].

The inflammatory cytokine TNF-α plays a central role in the pathogenesis of CIS-induced testicular injury [17,50]. Enhanced levels of the inflammatory marker TNF- α is associated with impairment of spermatogenesis and a decrease in testosterone levels [51,52]. It has been shown that an imbalance in TNF-α signaling can aggravate CIS-induced toxicity, considering the well-known facts related to TNF-α-induced change in the mitochondrial redox state and the associated damage to mitochondria [53,54]. Additionally, CIS-induced oxidative stress is reported to stimulate TNF-α production which is in turn could subsequently activate large network of pro-inflammatory cytokines such as IL-1β and IL-6 [55,56]. In the current study, we only focus on specific biomarkers which nevertheless incriminate their corresponding pathways: We detected a significant increase in testicular TNF-α level along with other inflammatory biomarkers of IL-1β and IL-6 in the CIS rat group, which are significantly mitigated by concomitant administration of different kinetin doses. Induction of the inflammatory cascades along with increased production of inflammatory cytokinin was suggested to be attributed to excessive ROS generated by CIS, which magnifies gonadotoxicity and spermatotoxicity [17,50]. Importantly, CIS-induced ROS generation also induces cellular stress and initiates a cascade of events, leading eventually to apoptosis [10,57]. Caspase-3 activation is produced, resulting from the sequence of events of the extrinsic phase of apoptosis which is defined by several characterized models including TNF-α and TNF- α receptors [58]. In rat testes, we reported that CIS significantly increased caspase-3 levels as an apoptotic executioner, which is responsible for the characteristic morphological changes of apoptosis that include membrane blebbing, cell shrinkage, and chromosomal DNA fragmentation. Nevertheless, we measured only the critical final executioner caspase and further studies should look at this in more detail, regarding the complete apoptosis pathway. Kinetin, in the present study, decreased the CIS-induced elevation in caspase-3 expression in a dose-dependent manner. Since the interplay between oxidative stress and inflammation is a hallmark of cell death [59], the decreased expression of caspase-3 in response to kinetin treatment may be secondary to its antioxidant and anti-inflammatory activities. Remarkably, significantly higher anti-inflammatory and antiapoptotic effects were observed in the CIS+ 1 Kn group compared to the CIS+ 0.25 Kn group. Additionally, the anti-inflammatory and antiapoptotic effects mediated by a dose of 1 mg/kg kinetin were more potent than those observed along in the Cis+ Vit C treatment group.

## 5. Conclusions

In conclusion, this study reveals that kinetin significantly preserved the integrity of rat testes, and maintained spermatogenesis after CIS toxicity. The testicular protective effect of kinetin was evident by reversing CIS-induced oxidative stress, as well as inflammatory and apoptotic effects in a dose-dependent manner.

## Figures and Tables

**Table 1:** Effect of different doses of kinetin and vitamin C on testicular content of malondialdehyde (MDA) and reduced glutathione (GSH) levels and activities of superoxide dismutase (SOD) and catalase activity in cisplatin-treated rats.

GROUPS	MDA (NMOLE/G TISSUE)	GSH (NMOLE/G TISSUE)	SOD (U/G TISSUE)	CATALASE (U/G TISSUE)
control	0.88 ± 0.018	2.7 ± 0.023	1.90 ± 0.027	2.22 ± 0.025
0.25 Kn	0.79 ± 0.016 *	2.917 ± 0.017 *	2.12 ± 0.029 *	2.44 ± 0.029 *
0.5 Kn	0.72 ± 0.010 *	3.023 ± 0.014 *	2.21 ± 0.012 *	2.53 ± 0.018 *
1 Kn	0.61 ± 0.020 *^#^	3.20 ± 0.027 *^#^	2.39 ± 0.027 *^#^	2.71 ± 0.029 *^#^
CIS	2.04 ±0.032 *	1.26 ± 0.039 *	0.78 ± 0.018 *	0.89 ± 0.014 *
CIS+ 0.25 Kn	1.82 ± 0.012 ^T^	1.44 ± 0.010 ^T^	0.96 ± 0.010 ^T^	1.24 ± 0.015 ^T^
CIS+ 0.5 Kn	1.67 ± 0.008 ^T^	1.52 ± 0.014 ^T^	1.06 ± 0.10 ^T^	1.26 ± 0.008 ^T^
CIS+ 1 Kn	1.58 ± 0.012 ^Tф^	1.74 ± 0.036 ^Tф^	1.34 ± 0.017 ^Tф^	1.57 ± 0.019 ^Tф^
CIS+ VitC	1.45 ± 0.032 ^Tα^	1.68 ± 0.035 ^T^	1.19 ± 0.043 ^Tα^	1.31 ± 0.045 ^Tα^

Data are represented as mean ± SEM; *, ^#^, ^T^, ^ф^ and ^α^ are significant differences from control, 0.25 Kn, CIS, CIS+ 0.25 Kn and CIS+ 1 Kn groups, respectively, at *p* < 0.05 where *n* = 5–6. Kn, kinetin; CIS, cisplatin; VitC, vitamin C.

## Data Availability

Data is contained within the article.

## Abbreviations

ASCO	American Society of Clinical Oncology
ASRM	American Society for Reproductive Medicine
CIS	Cisplatin
EDTA	Ethylene diamine tetra acetic acid
GnRH	Gonadotrophin-releasing hormone
GSH	Reduced glutathione
H&E	Hematoxylin–eosin
IL-1β	Interleukin-1 beta
IL-6	Interleukin-6
Kn	Kinetin
PBS	Phosphate buffer saline
PVDF	Polyvinylidene fluoride
ROS	Reactive oxygen species
SOD	Superoxide dismutase
StAR	Steroidogenic acute regulatory protein
TNF-α	Tumor Necrosis Factor alpha
VitC	Vitamin C
